# A pre-treatment comparison of referral pathways to guided ICBT for depression and anxiety disorders - A naturalistic study in routine clinical care

**DOI:** 10.3389/fdgth.2026.1633352

**Published:** 2026-03-18

**Authors:** Jill Bjarke, Marit Knapstad, Rolf Gjestad, Reidar Nævdal, Kjersti Skare, Tine Nordgreen

**Affiliations:** 1 Division of Psychiatry, Haukeland University Hospital, Bergen, Norway; 2 Department of Global Public Health and Primary Care, University of Bergen, Bergen, Norway; 3 Department of Health Promotion, Norwegian Institute of Public Health, Bergen, Norway; 4 Centre for Research and Education in Forensic Psychiatry, Haukeland University Hospital, Bergen, Norway; 5 Department of Biological and Medical Psychology, Faculty of Psychology, University of Bergen, Bergen, Norway

**Keywords:** anxiety disorders, depression, general practitioner referral, guided internet-delivered cognitive behavioral therapy, self-referral, specialized mental health care

## Abstract

**Introduction:**

Self-referral to therapist-guided internet-delivered cognitive behavioral therapy (guided ICBT) is increasingly being implemented in specialized mental healthcare settings to reduce barriers to care. Little is known about the characteristics of patients who access treatment through this pathway compared to the traditional referral pathway from general practitioner (GP). This study aims to compare demographic characteristics, socioeconomic and social factors between GP- and self-referred patients receiving guided ICBT for depression and anxiety disorders.

**Method:**

Naturalistic cross-sectional study comparing pre-treatment characteristics between GP- and self-referred patients in a specialized routine care ICBT clinic.

**Results:**

The study included 460 patients, 305 GP-referred, 155 self-referred. We found statistically significant differences between referral pathways in the socioeconomic factors; educational level (*P* < .001), employment status (*P* = .002), source of income (*P* = .004), social support (*P* = .029), types of healthcare utilization 6 months prior to treatment (*P* < .001) and information source about the treatment (*P* < .001). We found no difference in pre-treatment symptom level or basic demographics. Multiple logistic regression reduced the number of findings.

**Discussion:**

Different referral pathways potentially attract distinct patient populations, with self-referred patients being more likely to report greater socioeconomic resources than GP-referred. GP-referral remains crucial for individuals who may need more structured guidance to navigate healthcare systems. To promote equitable access, referral strategies should be tailored to reach those less likely to self-refer. Offering two pathways may support broader access to specialized mental healthcare. Future studies should further explore these identified statistical differences to optimize referral systems and ensure equitable access to mental healthcare for all in need.

## 1 Introduction

Mental disorders are leading causes of the global burden of disease (1). Depression and anxiety disorders are ranked among the most prevalent disorders (2, 3), affecting more females than males (1). Treatment is vital to reduce burden on the individual and societal level, and to prevent mental health problems from becoming chronic (4). However, access to mental healthcare is limited due to individual and systemic barriers (5).

At an individual level, barriers such as stigma and negative attitudes towards help-seeking can hinder individuals from seeking the support they need (6). Additionally, limited mental health literacy can prevent individuals from recognizing symptoms of mental health disorders or understanding the treatment options available to them (6, 7). At the system level, a barrier such as a shortage of mental health professionals (8) contribute to the overall treatment gap and unequal access to timely diagnosis and treatment (9). In addition, the general practitioners (GP) can also be a system level barrier due to their role as gatekeepers, managing referrals from primary care to specialized healthcare services (10). While this gatekeeping can contribute to delays and barriers in accessing specialized care, GPs also play a crucial role as “gate openers,” guiding patients through the healthcare system, facilitating appropriate and timely treatment (11). Although some individuals rely on their GP to facilitate access to treatment, others seek care independently and do not require GP guidance (12, 13). To address one of the system level barriers in specialized mental healthcare, self-referral has been proposed as a way to improve access to care (13, 14).

Self-referral has been established in the primary mental healthcare services for over two decades. It is a central component of the British Improving Access to Psychological Therapies (rebranded: NHS Talking Therapies) initiative (15). As first discussed by Brown et al. (13), self-referral was one of several measures introduced to reduce barriers and improve access to face-to-face and digital evidence-based psychological therapy. Through this system, individuals with self-identified mental health concerns can directly access services without requiring a GP-referral. This model is also implemented into primary care in Norway through the Prompt Mental Health Care (PMHC) program (16).

Self-referral to specialized routine mental health care is scarce, but the pathway has been implemented in Sweden, primarily through the Internet Psychiatry Clinic (17). However, the utilization of the self-referral pathway in specialized mental health services has received limited research attention, with only a handful of studies addressing patterns of use (18, 19).

Studies directly comparing GP- and self-referred patients in primary and specialized mental healthcare report mixed findings. A study of two similar digital mental health services within primary care in Australia (20), found that GP-referred patients had statistically higher pre-symptom levels of depression and anxiety than self-referred. However, the authors emphasized that self-referred individuals still reported higher symptom levels than those referred by GPs to ordinary face-to-face treatments. Due to the study's set up, GP-referred patients were more often males, significantly older, and less likely to have higher education and being employed than the self-referred (20). In contrast, a Danish study among patients with severe health anxiety within specialized healthcare found that self-referred patients scored significantly higher on measures of pre-treatment health anxiety, emotional distress, and somatic symptoms than the GP-referred (18). Self-referred patients were also, on average, 3.4 years older and had slightly higher educational levels (18). Additionally, a Swedish study examining mental disorders among young adults found that self-referrers to specialized mental healthcare exhibited higher pre-treatment symptom severity and more comorbid conditions compared to patients referred by GP and other nonpsychiatric referrals. This study found no differences in the basic demographics gender and age, and did not provide detailed information on educational level between the referral pathways (19). Another Swedish study found that various recruitment sources such as newspaper ads or mental health webpages, attract individuals with different symptom levels. However, the authors emphasize that further research in other countries is needed to establish patterns across different healthcare systems (21).

The literature above suggests that different pathways attract different patient populations. However, concerns about equity in self-referral pathways were raised early on, notably by Mathers and Mitchell (22) who hypothesized that self-referral to psychological therapy may favor more articulate, middle-class individuals with mild to moderate symptoms, and thereby potentially limiting access for already disadvantaged populations. This concern is supported by a recent systematic review, indicating that self-referral pathways can widen health disparities, by primarily attracting white, younger, well-educated, and less deprived women (23). However, while some argue that self-referral might attract individuals with minor concerns, the literature comparing clinical symptoms between GP- and self-referral to psychological treatment and mental health care offers little support for this claim (18–20). Since referral pathways may be linked to socioeconomic and social factors that affect access to care (23), understanding these differences is important for assessing whether self-referral increases accessibility for diverse groups or primarily benefits those with greater resources.

Moreover, referral pathways may also affect the implementation of novel treatments. Given this, the role of GPs as referral agents may significantly impact the adoption of new treatment formats. Skepticism among GPs towards integrating new methods and research findings into routine care has been identified as a barrier to the uptake of new treatments (24). Similarly, a recent systematic review report GP's skepticism towards digital treatment as a limitation for its uptake (25). In this context, the underutilization of a novel evidence-based treatment such as guided internet-delivered cognitive behavioral therapy (guided ICBT) serves as an example (14, 26, 27). More specifically, guided ICBT is a treatment found to yield treatment outcomes no different than that of face-to-face CBT (28). Guided ICBT is the first-line treatment for mild to moderate depression and anxiety disorders in several guidelines and national recommendations (29–31).

In sum, there are examples of clinical practices in specialized mental healthcare where both GP-referral and self-referral are used as pathways to care. Findings on differences in pre-treatment clinical symptoms between patients entering care through these referral pathways are mixed, and less is known about the patients’ differences in demographic and social characteristics. While some studies report pre-treatment characteristics, no prior research has focused specifically on comparing demographic, socioeconomic and social profiles across GP- and self-referral pathways in specialized mental healthcare. In line with previous research, we expect that self-referred patients will report higher educational levels, be more likely to be employed, and report higher levels of social support compared to the GP-referred.

### 1.1 Aim

This naturalistic study explores differences in pre-treatment characteristics across referral pathways by comparing GP-referred and self-referred patients receiving guided ICBT in specialized routine care mental health services. Specifically, we compare patients with depression and anxiety disorders, focusing on demographic characteristics, socioeconomic factors, and social support. In line with previous concerns about the demographic profile of those utilizing self-referral, we expect to observe differences that may shed light on the types of patients reached through each referral pathway.

## 2 Method

### 2.1 Design

This was a naturalistic cross-sectional study using data from a single pre-treatment assessment and conducted in a specialized routine care mental health clinic. The study compared two groups of patients: those referred by general practitioners (GP-referred) and those who self-referred, all of whom received guided internet-delivered cognitive behavioral therapy (guided ICBT) for moderate depression, panic disorder, or social anxiety disorder.

### 2.2 Setting

Participants were recruited from the eCoping clinic at Haukeland University Hospital in Bergen, Norway. Since 2013, the eCoping clinic has provided guided ICBT as part of its routine care for panic disorder (PD) (32) and social anxiety disorder (SAD) (33). Guided ICBT for moderate depression was introduced in 2015 (34). For the current study we present data collected at pre-treatment only. Data includes demographic information and information on the use of healthcare services covering 6 months prior to treatment start. GP-referred patients were enrolled between September 2014 and May 2019, while self-referred patients were enrolled between September 2016 and May 2019. Thus, the majority of both groups were recruited during an overlapping period (2016–2019), and all patients were assessed using identical inclusion criteria and procedures.

### 2.3 Two referral pathways to treatment

In Norway's specialized mental healthcare services, specialists assess patients’ symptoms and impairments to determine if they meet the criteria for legal access to this level of care (35). Primary healthcare services in Norway are for patients with mild to moderate conditions, while specialized healthcare is reserved for those with moderate and severe conditions. In the eCoping clinic, specialists in clinical psychology reviewed all referrals in accordance with the national priority guidelines to determine eligibility for specialized healthcare treatment (35).

#### 2.3.1 The established pathway

Patients referred by GPs contacted their GP who assessed symptom severity and impairment to determine if specialized mental healthcare services were necessary. If so, the GP initiated the referral to a specialist mental health outpatient clinic, or directly to the eCoping clinic. If the provider at the outpatient clinic determined that eCoping treatment was suitable or more appropriate than traditional face-to-face therapy, the patient was redirected to the eCoping clinic. These redirected patients are also categorized as GP-referred. GP-referred patients were enrolled in the study between September 2014 and May 2019.

#### 2.3.2 The novel pathway

Patients who self-referred contacted the eCoping clinic directly by emailing their contact details to an address provided on the eCoping website. An eCoping therapist conducted a telephone-based clinical interview to assess symptom severity and determine the eligibility of treatment at the specialist level. If so, the eCoping therapist initiated a referral based on the interview. These patients are categorized as self-referred. Self-referred patients were enrolled in the study between September 2016 and May 2019.

### 2.4 Inclusion and exclusion

Inclusion criteria for all study patients in the eCoping study were: 1) being 18 years of age or older, 2) if using antidepressants, being on a stable dosage over the previous four weeks, 3) being fluent in oral and written Norwegian. Exclusion criteria were: 1) current suicidal ideation, 2) current psychosis, 3) current substance abuse, 4) using benzodiazepines daily, 5) immediate need of other treatment, and 5) no access to the internet. Patients who were too mild or too severely ill were redirected elsewhere in the system, while patients who met the treatment criteria received an appointment for a face-to-face consultation with an ICBT therapist.

### 2.5 Pre-treatment procedure

All patients granted therapy participated in a face-to-face consultation including a structured clinical diagnostic interview using the 6th version of the Mini-International Neuropsychiatric Interview (MINI) (36). In cases of an unclear diagnosis, the therapists would do further assessment in accordance with their clinical discretion. Included patients were diagnosed with either moderate depression, PD, or SAD. Written informed consent was obtained from all patients prior to data collection. All self-report symptom measures and demographic questionnaires in the present study were administered via the internet and collected at pre-treatment. All digitalized versions of the assessment tools used in the eCoping study had previously been validated for their psychometric properties (37).

### 2.6 Outcomes

#### 2.6.1 Clinical symptoms

To expand the overall sample size and enhance the statistical power of the analyses (38), we created a harmonized primary outcome by merging the three diagnosis-specific outcomes and transforming their total scores: the Montgomery Åsberg Depression Rating Scale, Self-rating version (MADRS-S) (39), the Body Sensation Questionnaire (BSQ) (40), and the Social phobia scale (SPS) (41). The harmonization rescales each of the three outcome scores to a 0–100 range (see formula).

$$H=\frac{score-MIN}{MAX-MIN}*100$$

This standardization of total scores facilitates comparison of symptom reduction across diagnoses by combining diagnosis-specific measures into a single outcome of overall symptom burden, without altering the within-scale meaning. The harmonized outcome score has been sensitivity tested in two previous publications on the eCoping study (42, 43), where diagnosis-specific symptom trajectories and percentage reductions closely matched those observed in the harmonized score. These converging patterns support the methodological appropriateness of using a harmonized transdiagnostic outcome. However, formal psychometric validation at the item level could not be done, since no patients could have information on all three outcome variables. Consequently, internal reliability could not be assessed for the harmonized outcome measure.

Patients with depression were measured with the MADRS-S (39). This instrument assesses depression symptoms during the past 3 days using 9 items scored 0–6 with answer alternatives varying between items, where higher scores indicate more severe depression (total score range: 0–54). MADRS-S has been found sensitive to change (39, 44), with high correlations between expert ratings and self-reports (39). Internal consistency measured at pre-treatment with Cronbach's alpha yielded .77 for patients with GP-referral and .82 for self-referred patients.

Patients with panic disorder (PD) were measured with the BSQ (40). The instrument assesses intensity of fear or anxiety over the past week. The BSQ comprises 16 items rated on a 5-point Likert scale where higher scores indicate a higher level of fear and sensitivity to bodily sensations (total score range: 16–80). The BSQ has previously been found sensitive to symptom change during treatment (40). Internal consistency measured pre-treatment with Cronbach's alpha yielded .84 for patients with GP-referral and .88 for self-referred patients.

Patients with social anxiety disorder (SAD) were measured with the SPS (41). The instrument assesses the past week, entailing 20 questions rated on a 5-point Likert scale, where higher scores indicate higher anxiety (total score range: 0–100). The SAD is reliability and validity tested (41), and distinguishes individuals diagnosed with SAD from both healthy controls and individuals with other anxiety disorders (41). Internal consistency measured pre-treatment with Cronbach's alpha, yielded .91 for GP-referrals and .93 for self-referred patients.

#### 2.6.2 Demographic characteristics, socioeconomic, and social factors

Demographic factors were assessed with a questionnaire developed for this study to provide basic demographic information, including gender, age, relationship status, educational level, employment status, source of income, and level of social support, though not all items were necessarily completed. The questionnaire also gathered information on the number of years the patients had experienced symptoms of the current disorder. Additionally, patients were asked from what information source they learnt about the eCoping treatment.

Socioeconomic factors were assessed with the Treatment Inventory of Costs in Patients with psychiatric disorders (TiC-P) (45). The TiC-P is a questionnaire designed for adults with mental disorders measuring medical costs and productivity losses (45). TiC-P has a two-part structure, with the first part comprising 14 structured yes/no questions on the use of relevant medical resources. We computed the total use of health care services 6 months prior to treatment start by merging all the different service types of the patients reported to have utilized. The second part of the TiC-P consists of the Short Form-Health and Labour Questionnaire (SF-HLQ), a general tool to gather information on productivity losses related to health issues (45). The SF-HLQ is a short version of the Health and Labour Questionnaire (46), assessing work absenteeism. The TiC-P is has demonstrated feasibility, reliability, and satisfactory construct validity, making it a valuable tool for economic evaluations in mental healthcare (45). The current study used a 6-month recall period; thus, using an amended version of the questionnaire.

Social factors were assessed with a questionnaire measuring different aspects of functional and structural support. The questionnaire was designed by the research group affiliated with the eCoping study. The questionnaire entails 7 questions where higher scores indicate higher levels of social support (total score range: 0–27). Given support for a unidimensional factor structure, reporting the total score is most appropriate. Internal consistency measured by Cronbach's alpha, yielded .81 for GP-referrals and .79 for self-referred patients.

### 2.7 Statistics

Descriptive statistics include percentages, means, and standard deviations. Bivariate analyses included cross-tabulations of categorical variables using chi-square tests, ordinal regression of ordinal outcome variables, and t-tests for continuous outcome variables. We examined whether the observed bivariate associations were sensitive to adjustment in multivariable models. Multiple logistic regression analyzed associations between the outcome variable referral pathway and multiple predictors: demographic and socioeconomic information (Tables 1, 2), health care utilization 6 months prior to treatment start (Table 3), and the information source from where patients had learned about the treatment (Table 4). These four regression models were integrated into one main model. Explained variance was estimated with Nagelkerke. Temporal difference in inclusion period was controlled for by restricting analyses to 2016–2019, the main model was re-estimated on the restricted time interval. Logistic regression results include odds ratio (OR) and 95% confidence intervals (CI). Effect sizes (ES) for differences between GP- and self-referred patients were reported using Cohen's *d*, providing a standardized measure based on pooled standard deviations (47). Internal consistency was assessed with Cronbach's alpha. All analyses were conducted using IBM SPSS version 29 (48). The primary analyses assume missingness completely at random (MCAR) (49). A sensitivity analysis using multiple imputation (MI) under the missing at random (MAR) assumption was conducted to evaluate the impact of missing data.

**Table 1:** Observed pre-treatment symptom level and years of symptom complaints.

Outcome	GP-referred *N* = 305			Self-referred *N* = 155			*P*	*ES*
	*N*	*Mean*	*SD*	*N*	*Mean*	*SD*		
Pre-treatment symptom level^a^	289	46.0	17.5	151	43.9	18.6	.235	0.12
Years with symptoms	279	10.4	9.6	147	9.8	9.4	.571	0.06

GP-referred, referred by general practitioner; *N*, number of patients; SD, standard deviation.

^a^ Harmonized outcome: harmonization of MADRS-S, BSQ, and SPS. MADRS-S, Montgomery Åsberg Depression Rating Scale, Self-rating version; BSQ, Body Sensation Questionnaire; SPS, Social Phobia Scale.

**Table 2:** Demographic characteristics, socioeconomic and social factors by referral pathway.

Demography	GP-referred *N* = 305	Self-referred *N* = 155	*P*	*ES*
Gender: *N* (%)				
Female	185 (60.7%)	106 (68.4%)	.099	0.15
Male	120 (39.3%)	49 (31.6%)		
Age, mean (SD)	32.7 (11.4)	32.0 (10.3)	.491	0.07
Relationship status: *N* (%)				
Married/cohabitant	142 (50.0%)	83 (55.0%)	.324	0.09
Single	142 (50.0%)	68 (45.0%)		
Parental status: *N* (%)				
Yes	187 (64.9%)	104 (68.9%)	.406	0.08
No	101 (35.1%)	47 (31.1%)		
Education: *N* (%)				
Primary/Secondary level	189 (65.6%)	64 (42.4%)	<.001	0.46
University level	99 (34.4%)	87 (57.6%)		
Do you hold a job^a^: *N* (%)				
Yes	138 (53.7%)	94 (69.6%)	.002	0.33
No	119 (46.3%)	41 (30.4%)		
Currently on sick leave				
Yes	50 (36.2%)	32 (34.0%)	.732	0.04
No	88 (63.8%)	62 (66.0%)		
Source of income				
Home - Pension - Student^b^	39 (33.1%)	24 (60.0%)	.004^c^	0.45
Unemployed	13 (11.0%)	5 (12.5%)		
Welfare benefits	15 (12.7%)	1 (2.5%)		
Work assessment allowance	45 (38.1%)	8 (20.0%)		
Disability pension	6 (5.1%)	2 (5.0%)		
	N, M (SD)	N, M (SD)		
Social support – Total	288, 21.3 (4.6)	151, 22.3 (4.4)	.029	0.22

*N*, number of patients; SD, standard deviation; ES, effect size; GP-referred, referred by general practitioners.

^a^ Do you hold a job even if you currently are on sick leave?

^b^ Homemaker or pensioner or student.

^c^ *P*-value and ES based on ordinal regression.

**Table 3:** Health service use past 6 months by referral pathway.

Health services	GP-referred *N* = 305		Self-referred *N* = 155		*P*	*ES*
	*N*	*%*	*N*	*%*		
Public emergency room	57	22.3	26	19.3	.489	0.03
School healthcare service	16	6.3	6	4.5	.471	0.10
Industrial healthcare physician	7	2.7	7	5.2	.212	0.11
Physiotherapist	67	26.1	31	23.1	.525	0.10
Private practice psychologist	25	9.8	29	21.5	.001	0.11
Private practice psychiatrist	5	2.0	2	1.5	.738	0.03
Community based psychologist	36	14.1	18	13.3	.831	0.02
Community based psychiatric service	28	11.0	6	4.4	.029	0.22
Psychologist - psychiatrist outpatient clinic	133	52.2	24	17.8	<.001	0.71
Psychologist - psychiatrist hospital	9	3.5	1	0.7	.097	0.17
Community outpatient team	14	5.5	2	1.5	.058	0.19
Outpatient treatment psychiatric hospital	6	2.3	0	0.0	.073	0.18
Admitted to psychiatric hospital	3	1.2	0	0.0	.207	0.13
Total use of healthcare services	222	76.8	94	61.8	<.001	0.33

GP-referred, referred by general practitioner; *N*, number of patients; ES, effect size.

**Table 4:** Where did you learn about eCoping?

Learned about eCoping from:	GP-referred *N* = 305		Self-referred *N* = 155		*P* ^a^	*ES*
	*N*	*%*	*N*	*%*		
Outpatient clinic – psychologist - psychiatrist	139	56.7	14	9.3	<.001	1.14
General Practitioner	57	23.3	34	22.5		
Family or friend	10	4.1	23	15.2		
Internet or media	21	8.6	53	35.1		
Community psychiatric service	9	3.7	4	2.6		
Psychology service at the university	9	3.7	23	15.2		

GP-referred, referred by general practitioner; *N*, number of patients; ES, effect size.

^a^ Difference in distributions between all categories.

## 3 Results

This study reports data from 460 patients, 2/3 were GP-referred and 1/3 self-referred.

We found no differences in pre-treatment clinical symptom level between the two referral pathways or in the number of years the patients had experienced their symptoms (Table 1). A multiple logistic regression, with referral pathway as outcome, and pre-treatment symptom level (b_1_), years with symptoms (b_2_), age (b_3_) and gender (b_4_) as predictors, showed no statistically significant relations (OR_1_ = 0.99, *p* = .150, CI = 0.98–1.00; OR_2_ = 1.00, *p* = .972, CI = 0.98–1.02; OR_3_ = 1.00, *p* = .750, CI = 0.98–1.02; OR_4_ = 1.53, *p* = .053, CI = 1.00–2.36). Nagelkerke R^2^ was 0.02.

We found no difference in the demographic characteristics gender, age, relationship, and parental status between the referral pathways (Table 2). In terms of socioeconomic and social factors, more self-referred patients reported university level education and being employed, while more GP-referred patients reported receiving welfare benefits. About one-third of the total sample was on sick leave in the past four weeks prior to treatment start, but there were no differences between the referral pathway groups in this regard. Self-referred patients reported higher levels of social support compared to GP-referred. The effect sizes revealed the largest differences between the two groups in terms of source of income and educational level, but the differences remained within in the “small to medium” range.

We regressed the outcome referral pathway on the variables in Table 2, except for the variables currently on sick leave*,* due to the low sample size, and the nominal variable source of income. Educational level was the only statistically significant predictor of referral pathway (OR = 1.76, CI = 1.26–2.46; *p* < .001). Patients with higher educational levels had 76% higher likelihood of being in the self-referred group than in the GP-referred group. Nagelkerke was R^2^ = 0.09. No other variables were found to be statistically significant predictors of outcome.

We found some statistically significant differences between referral pathway groups in the use of healthcare services during the 6 months prior to treatment start (Table 3). Overall, though the majority in both referral groups had been in contact with some forms of healthcare services, the total healthcare use was higher among the GP-referred compared to those who self-referred. GP-referred patients accessed mostly psychologists and psychiatrists at outpatient clinics, or community based psychiatric service within the public healthcare system. Whereas self-referred patients utilized private practice psychologists to a higher extent than the GP-referred. The largest difference (ES) was related to GP-referred patients utilizing psychologist or psychiatrist at outpatient clinics.

In the multiple logistic regression with all predictors of healthcare use in Table 3, a higher likelihood of being in the self-referred group was found for patients who had contact with a private practice psychologist during the past six months prior to treatment start (OR = 2.90, *p* = .002, CI = 1.50–5.61). However, a decreased likelihood of self-referral was found for those having contact with community outpatient team (OR = 0.26, *p* = .007, CI = 0.10–0.69) and/or having contact with psychologist or psychiatrist in an outpatient clinic (OR = 0.16, *p* < .001, CI = 0.10–0.27). The predictors outpatient treatment and admission to psychiatric hospital, and total use of healthcare services, were excluded due to low numbers and multicollinearity. Nagelkerke was R^2^ = 0.25.

Further, approximately 76% of GP-referred patients reported learning about the eCoping treatment through their GP or another healthcare provider at an outpatient clinic (Table 4), compared to around 32% of self-referred patients. More self-referred patients learned about the treatment from family or friends, discovered it through online searches or media, or were informed by the psychological healthcare service at the university. The differences in distribution of information sources were large (ES).

The regression model, examining the source of information about eCoping, found that learning about eCoping from family or a friend (OR = 3.97, *p* = .002, CI = 1.68–9.37), from media or the internet (OR = 4.33, *p* < .001, CI = 2.22–8.44), or from the psychological services at the university (OR = 3.87, *p* = .003, CI = 1.57–9.54) was associated with an increased likelihood of self-referral. The reference group for these relations was GP. Reduced likelihood for self-referral was found related to learning about eCoping from a psychologist, psychiatrist or other therapists at an outpatient clinic, (OR = 0.17, *p* < .001, CI = 0.08–0.34). Nagelkerke was R^2^ = 0.38.

In the overall model, where all eligible predictors from Tables 1–4 were analyzed, the relations with education (*p* = .380) and community outpatient team disappeared (*p* = .067). All other results remained (private practice psychologist, psychologist/psychiatrist outpatient clinic, learned about eCoping from: outpatient clinic (reduced), family/friend (increased), media/internet (increased), psychological services at the university (increased), reference group=GP). No additional relations were supported (see Supplementary Table S1). Nagelkerke was R^2^ = 0.51. When analyses were repeated in the patient subsample (*N* = 266) using data from the restricted 2016–2019 period, the results were virtually identical to those observed in the full sample (*N* = 460; Supplementary Table S2).

The prevalence of missing data was relatively low and found to be equally distributed between the two referral groups, except for the relation between referral groups and where the patients learned about eCoping from (Supplementary Table S3). The multiple imputation results of the total prediction model under the MAR assumption produced similar results as in the original results (Supplementary Table S4). However, the relation between referral group and learning about eCoping from family or friend relatively to GP, was no longer statistically significant.

## 4 Discussion

In this naturalistic cross-sectional pre-treatment study from routine clinical care in specialized mental health services, we found several statistically significant differences between GP- and self-referred patients in demographic, socioeconomic, and social factors, use of health services and information sources about the treatment. These results align with our expectations regarding educational level, employment status and social support, and suggest that referral pathways may be associated with distinct demographic patient profiles at group level. At the same time, the referral groups did not differ in pre-treatment symptom severity, illness duration, gender or age. Our results are partially in line with previous studies comparing the two referral pathways. To determine whether the group differences represent independent associations, we re-examined them using multivariate models. In these models, only a subset of the bivariate differences remained statistically significant, indicating that several observed group differences were not independently related to referral pathway. Moreover, results from the restricted 2016–2019 subsample closely mirrored the full-sample findings, suggesting that the temporal overlap between pathways did not meaningfully influence the overall pattern of associations.

### 4.1 Clinical symptoms

Two recent studies from our research group based on the same patient cohort examining treatment effectiveness and predictors of treatment outcomes, reported no differences in pre-treatment symptom severity or illness duration between GP-referred and self-referred patients (42, 43). The present study replicated these findings. This lack of difference in pre-treatment symptom levels contrasts with previous research in primary care settings, which found higher pre-treatment symptom levels in GP-referred patients compared to those who self-referred (20). However, the study sample partially originated from a clinic designated at reaching specific populations. Our result also differs from findings in specialized care, where self-referred patients have been found to report higher symptom levels (18), and more comorbid diagnoses than those referred by GPs (19). However, symptom severity in our study was assessed after the initial face-to-face consultation that included the diagnostic MINI interview. Thus, the lack of difference between the referral pathway groups likely reflects that the ICBT therapists conducting the assessments applied consistent inclusion criteria and did not favor one referral pathway over the other. The lack of difference may also reflect the symptom level required to be eligible for treatment at the specialized level (35), and the redirection of patients too far above or below this threshold.

### 4.2 Demographic characteristics

We found no difference between the two referral pathway groups when exploring the demographics: gender, age, relationship, and parental status. The prevalence of female patients between referral pathways in our study aligns with findings from other studies comparing patients by referral pathway (18, 20), and with the gender distribution found across systematic reviews of ICBT (50–52). This pattern reflects the well-established gender disparity in help-seeking behavior within the mental health field. While being female is positively associated with help-seeking (53), males tend to avoid or delay seeking help (7, 54, 55). Men with low mental health literacy are the least likely to perceive a need for mental healthcare and often avoid seeking such help (7). In contrast, women with high mental health literacy are the least likely to experience unmet mental healthcare needs (56). Access to care is shaped by patients’ ability to perceive a need and to act on it, and these abilities may be influenced by factors such as education and social support (57). Although we did not measure health literacy directly, these factors are conceptually connected to literacy. In this context, the gender imbalance in our study suggests that current referral pathways may not sufficiently address barriers experienced by men. It is crucial to develop targeted strategies to increase the treatment's visibility and encourage help-seeking among this group**.**

### 4.3 Socioeconomic and social factors

Our result that self-referred patients had higher educational levels compared to GP-referred, aligns with previous research comparing patients by referral pathway (18). A systematic review of referral pathways across healthcare settings also found that individuals with higher educational levels are more likely to self-refer, with one study indicating that higher education was the strongest predictor of self-referral (23). In addition, several studies have found higher educational levels among patients in ICBT clinics (58–60), suggesting that this treatment format may attract individuals with higher education, regardless of referral pathway. Higher educational level emerged as a strong predictor of self-referral in the multiple regression analyses. However, the association between educational level and referral pathway disappeared in the main model. This suggests that educational level may be correlated with other patient characteristics included in the model and therefore does not independently account for differences in referral pathway. Notably, education might be regarded as a more distal causal factor compared to many of the other factors examined. Some of these factors may even be on the causal pathway between educational attainment and referral pathway. However, exploring such mediation is beyond the scope of the current study.

In the bivariate results we found a difference in level of social support, with those who self-referred reporting higher levels than the GP-referred. This result contributes to the overall pattern suggesting the two referral pathways may reach and be utilized by distinct patient populations. Moreover, the higher level of social support reported among self-referring patients may be related to differences in social, cognitive, and material resources, which are often distributed along a socioeconomic gradient (61). Social support can also shape help-seeking behavior by strengthening self-efficacy, for instance through verbal encouragement (e.g., being prompted to consider treatment) and observational learning (e.g., witnessing others benefit from it) (62). The multivariate results did not confirm the group difference in social support, thus the findings should be interpreted with caution.

We found that self-referred patients were more likely to be employed than GP-referred, whereas the two groups did not differ in their level of reported sick leave in the four weeks prior to treatment start. Both bivariate results partially aligns with previous research, where GP-referred patients had higher unemployment rates than self-referred (20), though findings on sick-leave between GP- and self-referred patients have been mixed (18). The relation between employment status and self-referral in general also provides mixed findings, with some studies showing that employed individuals are more likely to self-refer, while others find no differences (23). The mixed findings were replicated in our multivariate analyses, where the predictive values of being employed disappeared.

The reported differences in sources of income between referral pathways in our study may likely reflect broader socioeconomic gradients. Patients reporting to rely on welfare benefits or work assessment allowances were more often GP-referred, whereas those reporting stable income sources (e.g., employment, student support) were more likely to belong in the self-referred group. This pattern suggests that financial security may enhance autonomy in navigating healthcare systems, while economic vulnerability increases reliance on structured referral processes. However, these results likely reflect broader socioeconomic advantages, as financial security often co-occurs with higher education, health literacy, and personal resources that facilitate healthcare navigation (63). The findings related to differences in receiving welfare benefits or work assessment allowances may also partly reflect the structured nature of the GP-referral pathway. Receiving welfare benefits requires contact with a GP, and some benefits additionally require attending active treatment (64). The reported differences in income sources between referral the pathways in our study may also be relevant for understanding health outcomes, as education shapes access to stable employment and financial security, which in turn are linked to better health outcomes (63).

We found that GP-referred patients overall reported to have used healthcare services more frequently than the self-referred in the 6 months prior to treatment start. This difference likely reflects the structured nature of the GP-referral pathway, where the patients often underwent multiple steps and were redirected from an outpatient clinic before reaching the eCoping clinic. Moreover, the logistic regression showed that GP-referred patients’ higher overall healthcare utilization was not an independent predictor when specific service types were considered. Our adjusted analyses indicate that it is specifically contact with public mental-health services, such as psychologists or psychiatrists at outpatient-clinic and community outpatient team that independently predicts GP-referral, rather than a general pattern of high service use. However, the use of community outpatient team did not retain its predictive value of GP-referral in the main model. In contrast, more self-referred patients reported to have consulted private psychologists, and this remained a strong independent predictor of self-referral. This pattern of health care utilization may be associated with greater autonomy and resource availability, such as higher education, social support, and financial security. These two distinct patterns, public service use predicting GP-referral and private psychology use predicting self-referral, suggest that socioeconomic factors may be associated with both the choice of referral pathway and specific types of healthcare utilization rather than overall volume alone. From a service perspective, this raises important questions. While self-referral may potentially streamline access and reduce reliance on public health resources, it could inadvertently widen disparities if individuals with fewer personal resources remain dependent on their GPs as a gatekeepers to specialized mental health services (24). The strong predictive value of GP and public mental health services for GP-referral underscores this point. As our study did not assess comorbidities or functional impairments, we cannot determine whether the reported differences are driven by clinical complexity or systemic navigation patterns. Moreover, we did not assess the number of times each service was used. To our knowledge, no previous studies have examined patients’ overall healthcare status or their healthcare use prior to entering digital mental health treatment, highlighting the novelty of our findings. Our results add nuance by showing that it is the specific patterns of prior healthcare utilization, rather than the overall quantity of service use, that distinguish patients across referral pathways.

Due to the study's set up, more than half of the GP-referred patients reported to have learned about the treatment from a healthcare professional at an outpatient clinic. While half of the self-referred patients learned about the treatment from family, friends, the internet, or through media, these information sources were only reported by a small minority of the GP-referred (13%). This difference may reflect the higher educational and social support levels reported among self-referred patients. Nearly 20% of the total sample learned about the treatment from university psychology services, underscoring their role in raising awareness about available treatment options. According to the adjusted results, learning about the treatment from family, friends, media, internet or psychology services at the university increased the likelihood of self-referral. Interestingly, a similar proportion in both referral-groups had learnt about the treatment from their GP. However, as self-referrers contacted the eCoping clinic directly, this suggests an unexplored third pathway where GPs advised patients to contact the eCoping clinic without completing the referral process. This third pathway is previously described as GP-initiated self-referral (65). This approach is common in Prompt Mental Health Care (PMHC) (16). A study from the Swedish ICBT-clinic found that symptom severity corresponds with the source of information about ICBT treatment: active sources like online searches, mental health webpages, or clinic referrals attract patients with higher symptom levels, while passive sources like newspaper ads or information from friends or family attract those with lower symptom levels (21). Although these results differ from ours, the emphasis on recruitment sources influencing patient characteristics aligns with our findings that different referral pathways may attract distinct patient populations (21). More broadly, if internet-delivered mental health interventions are promoted primarily through digital channels, promoting them this way may unintentionally exclude individuals with limited digital access or skills. Digital promotion approaches risks reinforcing existing health inequities and widening the digital divide (66).

### 4.4 Limitations

This study has some limitations. First, as conducted in routine care, we have no data on individuals who were screened out, since data collection could not start before obtaining signed informed consent. This limits our ability to analyze differences between included and excluded patients across the two referral pathways but ensures that our findings reflect real-world clinical practice. Second, the study is exploratory, and although multivariable logistic regression was applied, the sample size relative to the number of predictors may limit the precision of estimations. However, the naturalistic routine clinical care context represents a strength of this study as a naturalistic design increases external validity, including the use of several established instruments with strong psychometric properties. To receive access to specialized mental healthcare services, all patients were diagnostically screened during their face-to-face consultation with the ICBT therapist. Those who did not meet the inclusion criteria, were judged too severely or not sufficiently ill, were excluded, resulting in a somewhat homogeneous sample. However, this strengthens internal validity by ensuring that comparisons are made within a well-defined clinical population. Another strength is that missing data in the variables were equally distributed between referral pathways. The results based on MI and the MAR assumption largely reproduced the results. However, one cannot rule out that missing data also may be missing not at random (MNAR). This assumption is not empirically testable (49).

### 4.5 Implications for healthcare services

Our results contribute to the overall pattern suggesting that the patients in our study represent two distinct populations divided by referral pathway. The results underscore the importance of implementing both GP- and self-referral pathways to specialized mental healthcare, as these options may enhance accessibility and expand the reach of services. Self-referrals may particularly appeal to individuals with higher levels of education and social support, while GP-referrals provide structured guidance for those who may face challenges navigating the healthcare system. To promote equitable access, outreach efforts should focus on individuals less likely to seek help independently, particularly those who may not fit the demographic profile typically associated with self-referral by highlighting the availability of accessible, effective services. An efficient referral system tailored for different patient groups is crucial not only for improving access to care but also for optimizing public healthcare resources and sustaining treatment capacity. Future research should explore the effectiveness of different referral pathways for diverse patients while addressing the social determinants that affect equity in mental health care access.

## 5 Conclusion

The present study comparing demographic, socioeconomic, and social factors across general practitioner (GP) and self-referral pathways to guided ICBT, suggests that these referral pathways may attract distinct patient populations. Although several differences initially appeared between the two groups, including differences in education, employment, and social support, only educational level remained a consistent distinguishing factor when considering the overall findings. These differences underscore the value of offering multiple referral pathways to ensure access for a broad range of individuals. While self-referral may lower barriers for some, GP-referral remains important in facilitating access to care for others. Further research is needed to identify how referral pathways can be optimized to reach individuals in need who might not otherwise access psychological treatment.

## Data Availability

The raw data supporting the conclusions of this article will be made available by the authors, without undue reservation.

## References

[1] GBD Mental Disorders Collaborators. Global, regional, and national burden of 12 mental disorders in 204 countries and territories, 1990–2019: a systematic analysis for the Global Burden of Disease Study 2019. Lancet Psychiatry. (2022) 9(2):137–50. 10.1016/S2215-0366(21)00395-3 35026139 PMC8776563

[2] Karyotaki E Efthimiou O Miguel C Genannt Bermpohl FM Furukawa TA Cuijpers P Internet-based cognitive behavioral therapy for depression: a systematic review and individual patient data network meta-analysis. JAMA Psychiatry. (2021) 78(4):361–71. 10.1001/jamapsychiatry.2020.4364 33471111 PMC8027916

[3] Van Dis EA Van Veen SC Hagenaars MA Batelaan NM Bockting CL Van Den Heuvel RM Long-term outcomes of cognitive behavioral therapy for anxiety-related disorders: a systematic review and meta-analysis. JAMA Psychiatry. (2020) 77(3):265–73. 10.1001/jamapsychiatry.2019.3986 31758858 PMC6902232

[4] Colizzi M Lasalvia A Ruggeri M. Prevention and early intervention in youth mental health: is it time for a multidisciplinary and trans-diagnostic model for care? Int J Ment Health Syst. (2020) 14:1–14. 10.1186/s13033-020-00356-9 32226481 PMC7092613

[5] Lattie EG Stiles-Shields C Graham AK. An overview of and recommendations for more accessible digital mental health services. Nat Rev Psychol. (2022) 1(2):87–100. 10.1038/s44159-021-00003-1 38515434 PMC10956902

[6] Schnyder N Panczak R Groth N Schultze-Lutter F. Association between mental health-related stigma and active help-seeking: systematic review and meta-analysis. Br J Psychiatry. (2017) 210(4):261–8. 10.1192/bjp.bp.116.189464 28153928

[7] Gough B Novikova I. Mental Health, Men and Culture: How do Sociocultural Constructions of Masculinities Relate to Men's Mental Health Help-Seeking Behaviour in the WHO European Region? Copenhagen: WHO (2020). 32716621

[8] Butryn T Bryant L Marchionni C Sholevar F. The shortage of psychiatrists and other mental health providers: causes, current state, and potential solutions. Int J Acad Med. (2017) 3(1):5–9. 10.4103/ijam.Ijam_49_17

[9] Lindberg MS Brattmyr M Lundqvist J Solem S Hjemdal O Roos E Is the Norwegian stepped care model for allocation of patients with mental health problems working as intended? A cross-sectional study. Psychother Res. (2024) 35:1–13. 10.1080/10503307.2024.2378017 39037043

[10] Hoff EH Kraft KB Moe CF Nylenna M Østby KA Mykletun A. The cost of saying no: general practitioners’ gatekeeping role in sickness absence certification. BMC Public Health. (2024) 24(1):439. 10.1186/s12889-024-17993-1 38347474 PMC10860288

[11] Ridd MJ Thompson MJ. Would primary care paediatricians improve UK child health outcomes? No. Br J Gen Pract. (2020) 70(693):196. 10.3399/bjgp20X709289 32217599 PMC7098524

[12] Andersson G Titov N. Advantages and limitations of internet-based interventions for common mental disorders. World Psychiatry. (2014) 13(1):4–11. 10.1002/wps.20083 24497236 PMC3918007

[13] Brown JS Boardman J Whittinger N Ashworth M. Can a self-referral system help improve access to psychological treatments? Br J Gen Pract. (2010) 60(574):365–71. 10.3399/bjgp10X501877 20423587 PMC2858533

[14] Folker AP Mathiasen K Lauridsen SM Stenderup E Dozeman E Folker MP. Implementing internet-delivered cognitive behavior therapy for common mental health disorders: a comparative case study of implementation challenges perceived by therapists and managers in five European internet services. Internet Interv. (2018) 11:60–70. 10.1016/j.invent.2018.02.001 30135761 PMC6084870

[15] Wakefield S Kellett S Simmonds-Buckley M Stockton D Bradbury A Delgadillo J. Improving access to psychological therapies (IAPT) in the United Kingdom: a systematic review and meta-analysis of 10-years of practice-based evidence. Br J Clin Psychol. (2021) 60(1):e12259. 10.1111/bjc.12259 32578231

[16] Knapstad M Nordgreen T Smith OR. Prompt mental health care, the Norwegian version of IAPT: clinical outcomes and predictors of change in a multicenter cohort study. BMC Psychiatry. (2018) 18(1):1–16. 10.1186/s12888-018-1838-0 30115041 PMC6097447

[17] Vernmark K Buhrman M Carlbring P Hedman-Lagerlöf E Kaldo V Andersson G. From research to routine care: a historical review of internet-based cognitive behavioral therapy for adult mental health problems in Sweden. Digital Health. (2024) 10:20552076241287059. 10.1177/20552076241287059 39381804 PMC11459524

[18] Hoffmann D Rask CU Hedman-Lagerlöf E Eilenberg T Frostholm L. Accuracy of self-referral in health anxiety: comparison of patients self-referring to internet-delivered treatment versus patients clinician-referred to face-to-face treatment. BJPsych Open. (2019) 5(5):e80. 10.1192/bjo.2019.54 31496462 PMC6737511

[19] Ramirez A Ekselius L Ramklint M. Mental disorders among young adults self-referred and referred by professionals to specialty mental health care. Psychiatr Serv. (2009) 60(12):1649–55. 10.1176/ps.2009.60.12.1649 19952156

[20] Staples LG Webb N Asrianti L Cross S Rock D Kayrouz R A comparison of self-referral and referral via primary care providers, through two similar digital mental health services in Western Australia. Int J Environ Res Public Health. (2022) 19(2):905. 10.3390/ijerph19020905 35055727 PMC8775987

[21] Lindner P Nyström MBT Hassmén P Andersson G Carlbring P. Who seeks ICBT for depression and how do they get there? Effects of recruitment source on patient demographics and clinical characteristics. Internet Interv. (2015) 2(2):221–5. 10.1016/j.invent.2015.04.002

[22] Mathers N Mitchell C. Are the gates to be thrown open? Br J Gen Pract. (2010) 60(574):317–8. 10.3399/bjgp10X484138 20423580 PMC2858526

[23] Harvey-Sullivan A Lynch H Tolley A Gitlin-Leigh G Kuhn I Ford JA. What impact do self-referral and direct access pathways for patients have on health inequalities? Health Policy. (2024) 139:104951. 10.1016/j.healthpol.2023.104951 38096622

[24] Ross J Stevenson F Lau R Murray E. Factors that influence the implementation of e-health: a systematic review of systematic reviews (an update). Implement Sci. (2016) 11(1):146. 10.1186/s13012-016-0510-7 27782832 PMC5080780

[25] Duffy D Richards D Hisler G Timulak L. Implementing internet-delivered cognitive behavioral therapy for depression and anxiety in adults: systematic review. J Med Internet Res. (2025) 27:e47927. 10.2196/47927 39874577 PMC11815312

[26] Molloy A Ellis DM Su L Anderson PL. Improving acceptability and uptake behavior for internet-based cognitive-behavioral therapy. Front Digit Health. (2021) 3:653686. 10.3389/fdgth.2021.653686 34713125 PMC8521972

[27] Vis C Schuurmans J Aouizerate B Atipei Craggs M Batterham P Bührmann L Effectiveness of self-guided tailored implementation strategies in integrating and embedding internet-based cognitive behavioral therapy in routine mental health care: results of a multicenter stepped-wedge cluster randomized trial. J Med Internet Res. (2023) 25:e41532. 10.2196/41532 36735287 PMC9938445

[28] Hedman-Lagerlöf E Carlbring P Svärdman F Riper H Cuijpers P Andersson G. Therapist-supported internet-based cognitive behaviour therapy yields similar effects as face-to-face therapy for psychiatric and somatic disorders: an updated systematic review and meta-analysis. World Psychiatry. (2023) 22(2):305–14. 10.1002/wps.21088 37159350 PMC10168168

[29] Helsedirektoratet. Ti tiltak for å redusere sykdomsbyrden og bedre folkehelsen (2018). Available online at: https://www.helsedirektoratet.no/rapporter/ti-tiltak-for-a-redusere-sykdomsbyrden-og-bedre-folkehelsen/Ti%20tiltak%20for%20%C3%A5%20redusere%20sykdomsbyrden%20og%20bedre%20folkehelsen%20(NCD).pdf/_/attachment/inline/fdeec3bc-0b2f-4370-9ed6-4dcbcd8dbe35:4b883ef837ea70e2dfd217c287163f2d1bc0d1b3/Ti%20tiltak%20for%20%C3%A5%20redusere%20sykdomsbyrden%20og%20bedre%20folkehelsen%20(NCD).pdf (Accessed January 07, 2025).

[30] National Institute for Health Care Excellence. Depression in adults: treatment and management (2022). Available online at: https://www.nice.org.uk/guidance/ng222/chapter/recommendations#treatment-manuals (Accessed January 07, 2025). 35977056

[31] Swedish National Board of Wellfare. Nationella Riktlinjer för Vård vid Depression och Ångestsyndrom. Stöd för Styrning och Ledning. The National Board of Wellfare Stockholm, Sweden (2016). Available online at: https://www.socialstyrelsen.se/globalassets/sharepoint-dokument/artikelkatalog/nationella-riktlinjer/2021-4-7339.pdf (Accessed January 07, 2025).

[32] Nordgreen T Gjestad R Andersson G Carlbring P Havik OE. The implementation of guided internet-based cognitive behaviour therapy for panic disorder in a routine-care setting: effectiveness and implementation efforts. Cogn Behav Ther. (2018) 47(1):62–75. 10.1080/16506073.2017.1348389 28714775

[33] Nordgreen T Gjestad R Andersson G Carlbring P Havik OE. The effectiveness of guided internet-based cognitive behavioral therapy for social anxiety disorder in a routine care setting. Internet Interv. (2018) 13:24–9. 10.1016/j.invent.2018.05.003 30206515 PMC6112093

[34] Nordgreen T Blom K Andersson G Carlbring P Havik OE. Effectiveness of guided internet-delivered treatment for major depression in routine mental healthcare-an open study. Internet Interv. (2019) 18:100274. 10.1016/j.invent.2019.100274 31890623 PMC6926287

[35] Helsedirektoratet. Prioriteringsveileder - psykisk helsevern for voksne (2015). Available online at: https://www.helsedirektoratet.no/veiledere/prioriteringsveiledere/psykisk-helsevern-for-voksne (Accessed June 19, 2024).

[36] Sheehan DV Lecrubier Y Sheehan KH Amorim P Janavs J Weiller E The Mini-International Neuropsychiatric Interview (MINI): the development and validation of a structured diagnostic psychiatric interview for DSM-IV and ICD-10. J Clin Psychiatry. (1998) 59(20):22–33. Available online at: https://psycnet.apa.org/record/1998-03251-004 (Accessed December 18, 2024). 9881538

[37] Carlbring P Brunt S Bohman S Austin D Richards J Öst L-G Internet vs. paper and pencil administration of questionnaires commonly used in panic/agoraphobia research. Comput Human Behav. (2007) 23(3):1421–34. 10.1016/j.chb.2005.05.002

[38] Zantvoort K Hentati Isacsson N Funk B Kaldo V. Dataset size versus homogeneity: a machine learning study on pooling intervention data in e-mental health dropout predictions. Digit Health. (2024) 10:20552076241248920. 10.1177/20552076241248920 38757087 PMC11097733

[39] Svanborg P Åsberg M. A new self-rating scale for depression and anxiety states based on the comprehensive psychopathological rating scale. Acta Psychiatr Scand. (1994) 89(1):21–8. 10.1111/j.1600-0447.1994.tb01480.x 8140903

[40] Chambless DL Caputo GC Bright P Gallagher R. Assessment of fear of fear in agoraphobics: the body sensations questionnaire and the agoraphobic cognitions questionnaire. J Consult Clin Psychol. (1984) 52(6):1090–7. 10.1037/0022-006x.52.6.1090 6520279

[41] Mattick RP Clarke JC. Development and validation of measures of social phobia scrutiny fear and social interaction anxiety. Behav Res Ther. (1998) 36(4):455–70. 10.1016/S0005-7967(97)10031-6 9670605

[42] Bjarke J Gjestad R Nordgreen T. Effectiveness of general practitioner referral versus self-referral pathways to guided internet-delivered cognitive behavioral therapy for depression, panic disorder, and social anxiety disorder: naturalistic study. JMIR Ment Health. (2025) 12:e68165. 10.2196/68165 40135415 PMC11962571

[43] Bjarke J Gjestad R Nordgreen T. Predicting the path to guided ICBT success across referral pathway for moderate depression and anxiety disorders. Internet Interv. (2026) 43:100907. 10.1016/j.invent.2026.100907 41624506 PMC12856857

[44] Fantino B Moore N. The self-reported Montgomery-Asberg Depression Rating Scale is a useful evaluative tool in major depressive disorder. BMC Psychiatry. (2009) 9:26. 10.1186/1471-244x-9-26 19473506 PMC2701427

[45] Bouwmans C De Jong K Timman R Zijlstra-Vlasveld M Van der Feltz-Cornelis C Tan SS Feasibility, reliability and validity of a questionnaire on healthcare consumption and productivity loss in patients with a psychiatric disorder (TiC-P). BMC Health Serv Res. (2013) 13(1):217. 10.1186/1472-6963-13-217 23768141 PMC3694473

[46] Van Roijen L Essink-Bot M-L Koopmanschap MA Bonsel G Rutten FF. Labor and health status in economic evaluation of health care: the health and labor questionnaire. Int J Technol Assess Health Care. (1996) 12(3):405–15. 10.1017/S0266462300009764 8840661

[47] Cohen J. Statistical Power Analysis for the Behavioral Sciences. New York: Routledge (2013).

[48] IBM Corp. IBM SPSS Statistics for Windows, version 29.0 (2023).

[49] Enders CK. Applied Missing Data Analysis. New York: Guilford Publications (2022).

[50] Børtveit L Dechsling A Sütterlin S Nordgreen T Nordahl-Hansen A. Guided internet-delivered treatment for depression: scoping review. JMIR Ment Health. (2022) 9(10):e37342. 10.2196/37342 36194467 PMC9579933

[51] Haller K Becker P Niemeyer H Boettcher J. Who benefits from guided internet-based interventions? A systematic review of predictors and moderators of treatment outcome. Internet Interv. (2023) 33:100635. 10.1016/j.invent.2023.100635 37449052 PMC10336165

[52] Oliveira C Pacheco M Borges J Meira L Santos A. Internet-delivered cognitive behavioral therapy for anxiety among university students: a systematic review and meta-analysis. Internet Interv. (2023) 31:100609. 10.1016/j.invent.2023.100609 36873307 PMC9982642

[53] Magaard JL Seeralan T Schulz H Brütt AL. Factors associated with help-seeking behaviour among individuals with major depression: a systematic review. PLoS One. (2017) 12(5):e0176730. 10.1371/journal.pone.0176730 28493904 PMC5426609

[54] Kazdin AE. Addressing the treatment gap: a key challenge for extending evidence-based psychosocial interventions. Behav Res Ther. (2017) 88:7–18. 10.1016/j.brat.2016.06.004 28110678

[55] Kuang K Wang N. Predictors and outcomes of help-seeking behaviors: a longitudinal investigation in mental illness. Health Commun. (2024) 39(9):1906–17. 10.1080/10410236.2023.2247157 37574866

[56] Blom S Lindh F Lundin A Burström B Hensing G Löve J. How gender and low mental health literacy are related to unmet need for mental healthcare: a cross-sectional population-based study in Sweden. Arch Public Health. (2024) 82(1):12. 10.1186/s13690-023-01228-7 38273389 PMC10809616

[57] Cu A Meister S Lefebvre B Ridde V. Assessing healthcare access using the Levesque's conceptual framework– a scoping review. Int J Equity Health. (2021) 20(1):116. 10.1186/s12939-021-01416-3 33962627 PMC8103766

[58] El Alaoui S Hedman E Kaldo V Hesser H Kraepelien M Andersson E Effectiveness of internet-based cognitive–behavior therapy for social anxiety disorder in clinical psychiatry. J Consult Clin Psychol. (2015) 83(5):902–14. 10.1037/a0039198 26009780

[59] Hedman E Ljótsson B Kaldo V Hesser H El Alaoui S Kraepelien M Effectiveness of internet-based cognitive behaviour therapy for depression in routine psychiatric care. J Affect Disord. (2014) 155:49–58. 10.1016/j.jad.2013.10.023 24238951

[60] Hedman E Ljótsson B Rück C Bergström J Andersson G Kaldo V Effectiveness of internet-based cognitive behaviour therapy for panic disorder in routine psychiatric care. Acta Psychiatr Scand. (2013) 128(6):457–67. 10.1111/acps.12079 23406572

[61] Adams J Mytton O White M Monsivais P. Correction: why are some population interventions for diet and obesity more equitable and effective than others? The role of individual agency. PLoS Med. (2016) 13(5):e1002045. 10.1371/journal.pmed.1001990 27046234 PMC4821622

[62] Bandura A. Self-efficacy mechanism in human agency. Am Psychol. (1982) 37(2):122–47. 10.1037/0003-066X.37.2.122

[63] Zajacova A Lawrence EM. The relationship between education and health: reducing disparities through a contextual approach. Annu Rev Public Health. (2018) 39(1):273–89. 10.1146/annurev-publhealth-031816-044628 29328865 PMC5880718

[64] Saunes IS Karanikolos M Sagan A. Norway: health system review. Health Syst Transit. (2020) 22(1):i–163. 32863241

[65] Jonker L Thwaites R Fisher SJ. Patient referral from primary care to psychological therapy services: a cohort study. Fam Pract. (2019) 37(3):395–400. 10.1093/fampra/cmz094 31829408

[66] Berardi C Antonini M Jordan Z Wechtler H Paolucci F Hinwood M. Barriers and facilitators to the implementation of digital technologies in mental health systems: a qualitative systematic review to inform a policy framework. BMC Health Serv Res. (2024) 24(1):243. 10.1186/s12913-023-10536-1 38408938 PMC10898174

[67] World Medical Association. 64th WMA General Assembly Fortaleza Brazil, October 2013. WMA Declaration of Helsinki–Ethical Principles for Medical Research Involving Human Subjects (2018). Available online at: https://www.wma.net/policies-post/wma-declaration-of-helsinki/ (Accessed January 11, 2024).

