# Supplementary material for: A pre-treatment comparison of referral pathways to guided ICBT for depression and anxiety disorders - A naturalistic study in routine clinical care
Source: Front Digit Health. 2026 Mar 18;8:1633352. doi: 10.3389/fdgth.2026.1633352 (PMC13038992; doi:10.3389/fdgth.2026.1633352)

**Supplementary Material**

**Supplementary Table 1: Total logistic regression model (N = 460)**


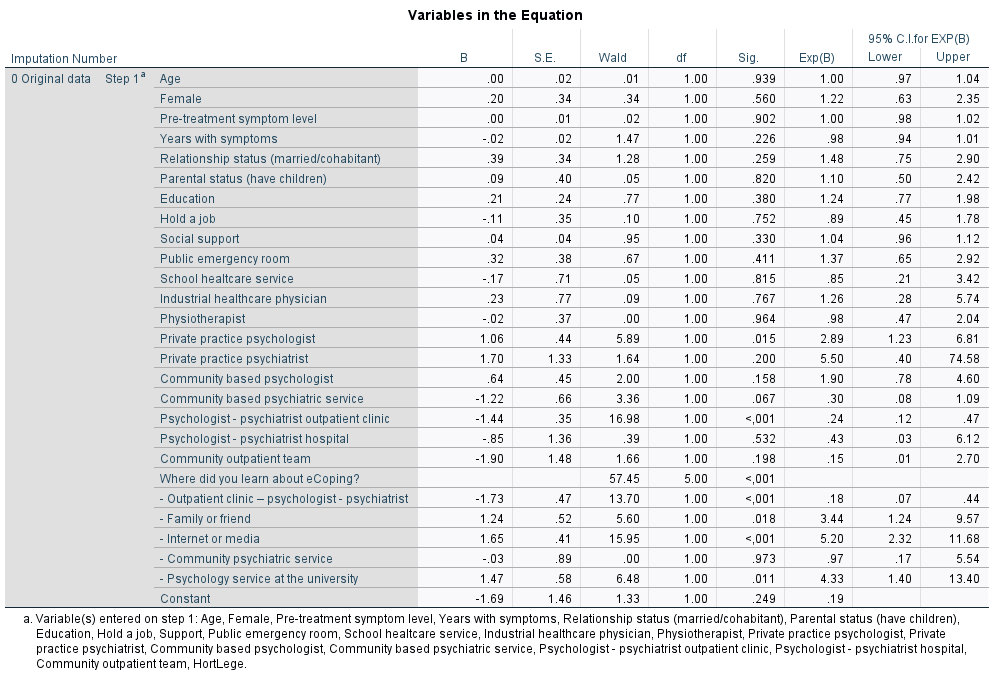


**Supplementary Table 2: Total logistic regression model: Patient sub-sample from the same inclusion period 2016 - 2019 (N = 266)**


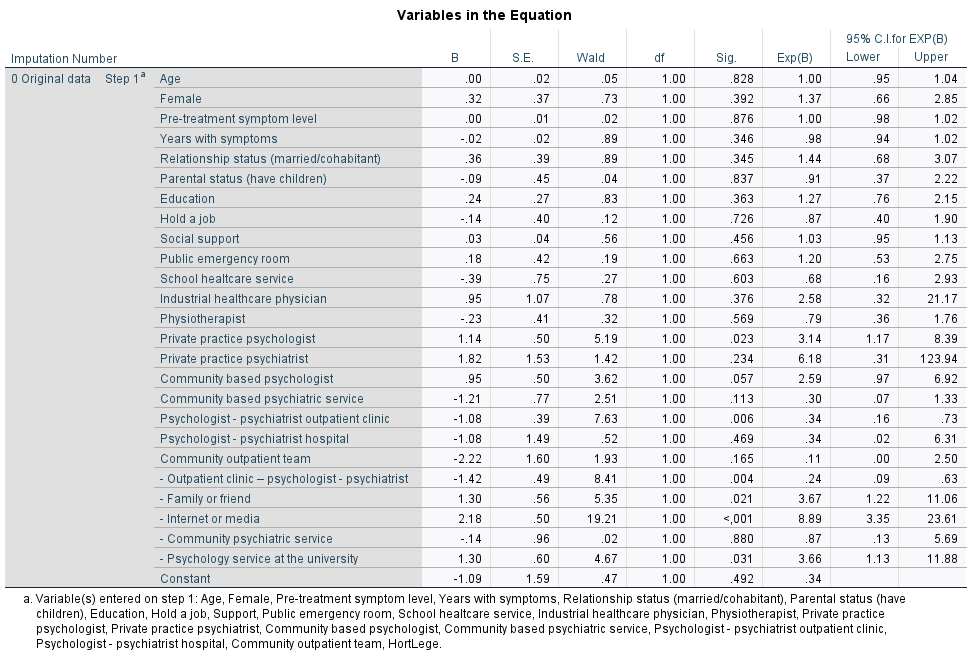


**Supplementary Table 3: Missing data in variables (N = 460)**

Percent missing in GP- and Self-referred groups

| Referral status: |  | GP |  | Self |  | Difference | |
| --- | --- | --- | --- | --- | --- | --- | --- |
|  |  | % |  | % |  | P-value(χ^2^) | r |
| Gender |  | 0 |  | 0 |  | - | - |
| Age |  | 0 |  | 0 |  | - | - |
| Pre-treatment symptom level (Harmonized) |  | 5.2 |  | 3.5 |  | .185 | .06 |
| Years with symptoms |  | 0 |  | 0 |  | - | - |
| Relationship status: Married / Cohabitant |  | 6.9 |  | 2.6 |  | .054 | -.09 |
| Parental status: Kids |  | 5.6 |  | 2.6 |  | .146 | -.07 |
| Education |  | 5.6 |  | 2.6 |  | .146 | -.07 |
| Job |  | 0 |  | 0 |  | - | - |
| Sick Leave |  | 10.5 |  | 11.0 |  | .876 | .01 |
| Source of income |  | 10.5 |  | 11.0 |  | .876 | .01 |
| Social support |  | 5.6 |  | 2.6 |  | .146 | -.07 |
| Public emergency room |  | 10.8 |  | 11.0 |  | .962 | .00 |
| School healthcare service |  | 10.8 |  | 11.6 |  | .798 | .01 |
| Industrial healthcare physician |  | 10.5 |  | 11.0 |  | .876 | .01 |
| Physiotherapist |  | 10.5 |  | 11.6 |  | .715 | .02 |
| Private practice psychologist |  | 11.1 |  | 11.0 |  | .954 | -.00 |
| Private practice psychiatrist |  | 10.8 |  | 11.0 |  | .962 | .00 |
| Community based psychologist |  | 11.1 |  | 11.0 |  | .954 | -.00 |
| Community based psychiatric service |  | 11.5 |  | 11.0 |  | .871 | -.01 |
| Psychologist - psychiatrist outpatient clinic |  | 11.1 |  | 11.0 |  | .954 | -.00 |
| Psychologist - psychiatrist hospital |  | 11.1 |  | 11.0 |  | .954 | -.00 |
| Community outpatient team |  | 10.8 |  | 11.0 |  | .962 | .00 |
| Day treatment psychiatric hospital |  | 10.8 |  | 11.0 |  | .962 | .00 |
| Admitted to psychiatric hospital |  | 10.8 |  | 11.0 |  | .962 | .00 |
| Total use of healthcare services |  | 10.5 |  | 11.0 |  | .876 | .01 |
| Where did you learn about eCoping? |  | 19.7 |  | 2.6 |  | <.001 | -.23 |

**Supplementary Table 4: Full logistic regression model based on Multiple Imputation (MI) (N = 460), imputed data sets = 50**


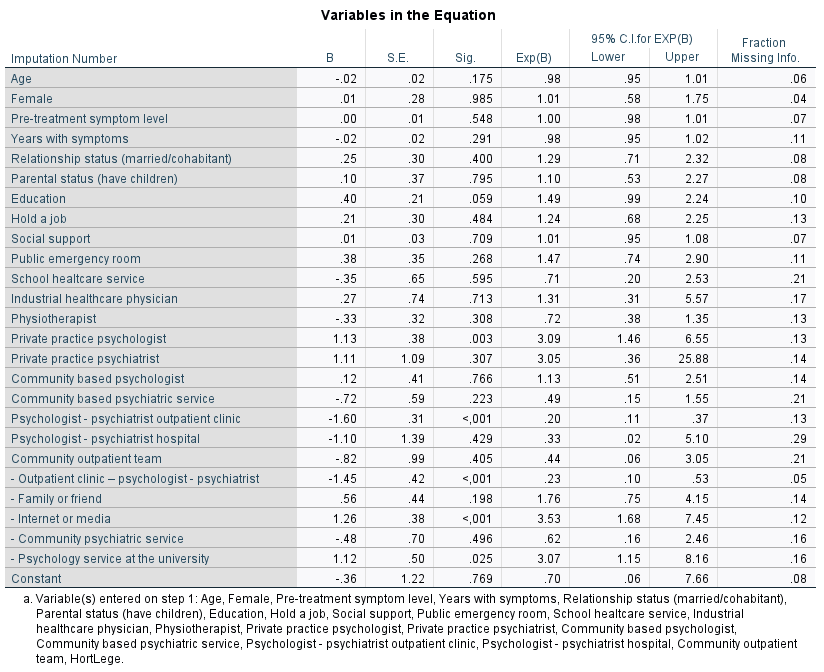

Supplement: Supplementary file 1 [file Datasheet1.docx]
